# A recombinant *Aspergillus oryzae* fungus transmitted from larvae to adults of *Anopheles stephensi* mosquitoes inhibits malaria parasite oocyst development

**DOI:** 10.1038/s41598-023-38654-0

**Published:** 2023-07-27

**Authors:** Leila Kianifard, Ab. Matteen Rafiqi, Osman Akcakir, Ahmed S. I. Aly, Peter F. Billingsley, Serdar Uysal

**Affiliations:** 1 Beykoz Institute of Life Sciences and Biotechnology, Bezmialem Vakif University, 34820 Istanbul, Turkey; 2 School of Science and Engineering, Al Akhawayn University, Ifrane, 53000 Morocco; 3 Sanaria Inc., 9800 Medical Center Dr., Suite A209, Rockville, MD 20850 USA

**Keywords:** Fungi, Parasitology, Disease prevention, Peptides

## Abstract

The control of malaria parasite transmission from mosquitoes to humans is hampered by decreasing efficacies of insecticides, development of drug resistance against the last-resort antimalarials, and the absence of effective vaccines. Herein, the anti-plasmodial transmission blocking activity of a recombinant *Aspergillus oryzae* (*A. oryzae*-R) fungus strain, which is used in human food industry, was investigated in laboratory-reared *Anopheles stephensi* mosquitoes. The recombinant fungus strain was genetically modified to secrete two anti-plasmodial effector peptides, MP2 (midgut peptide 2) and EPIP (enolase-plasminogen interaction peptide) peptides. The transstadial transmission of the fungus from larvae to adult mosquitoes was confirmed following inoculation of *A. oryzae*-R in the water trays used for larval rearing. Secretion of the anti-plasmodial effector peptides inside the mosquito midguts inhibited oocyst formation of *P. berghei* parasites. These results indicate that *A. oryzae* can be used as a paratransgenesis model carrying effector proteins to inhibit malaria parasite development in *An. stephensi*. Further studies are needed to determine if this recombinant fungus can be adapted under natural conditions, with a minimal or no impact on the environment, to target mosquito-borne infectious disease agents inside their vectors.

## Introduction

*Plasmodium*, which causes malaria is the most important vector-borne disease and is transmitted through the bite of infected anopheline mosquitoes^1^. Nearly half of the world's population lives in malaria-prone areas and more than 400,000 people die from malaria each year, most of them young children^1^. Despite continued management efforts, malaria control has had limited success, infection levels have plateaued, and eradication remains elusive. Undoubtedly, the current COVID-19 pandemic has indirect implications for malaria control. The rapid response to COVID-19 must inspire efforts to improve malaria control, otherwise there is a risk of an increase in the mortality caused by this pandemic, particularly among children, and undoing one of the most effective public health campaigns^2^. Consequently, there is an urgent need for practical alternatives to control malaria^1^.

Mosquitoes are the definitive hosts of malaria and many other vector-borne diseases. *Anopheles stephensi* is a major vector of malaria in Asia and has recently spread to the Horn of Africa^3^. In the mosquito, the parasite undergoes developmental, multiplication and maturation steps within the midgut^4^. The midgut may therefore be considered a prime target for intervention, such that competition between microbiomes in the gut may result in a particular species gaining the upper hand in the gut^5,6^. The mosquito microbiota can be derived from the environment at any of the several stages throughout the mosquito's life cycle and has important effects via interactions with mosquito biological processes, such as growth, immune responses, digestion, reproduction, and resistance to pathogens^7,8^. The species composition of the mosquito microbiome is thus very dynamic and diverse^9–11^.

Over recent years, much of the malaria research agenda has focused on drug or vaccine development. As the efficacy of insecticides and malaria treatments declines and due to the inadequacy of effective vaccines against mosquito-borne diseases, results from the most advanced malaria vaccine (RTS, S) suggest that it only reduces morbidity and is therefore inadequate in terms of a tool to achieve the goal of eradicating malaria^1^. The demand for more dynamic vector-targeted control approaches is increasingly developing^12^. Among recently proposed methods, paratransgenesis is a novel and multifaceted pathway approach that has been suggested as a potential means to control vector-borne diseases. This approach attempts to eliminate a pathogen from a vector community by genetically manipulating symbiotic organisms of the vector, such as bacteria, viruses, or fungi, by inducing the endosymbiont to produce antipathogenic effector molecules^13,14^. Microorganisms including viruses^15^, fungi^13,16^, and bacteria^17^ have been tested as paratransgenesis candidates in malaria vectors. To facilitate the approach, the microorganisms being used for paratransgenesis must be associated with the target vector population, must grow efficiently in commonly available and inexpensive media, and must be genetically modifiable in the laboratory. After genetic manipulation, they must remain similar to the wild type and must colonize and dominate within the vector efficiently and finally must be safe with respect to humans, the environment, and non-target animals^18^.

Most studies related to the paratransgensis approach have concentrated on mosquito midgut microbiota^8,19,20^. In this study, we focused on *Aspergillus oryzae* (*A. oryzae*), which is used to produce fermented foods and beverages such as sake and shoyu. In addition, it can be isolated from soils and plants and is not harmful to humans or animals. *A. oryzae* is a microorganism that is generally regarded as safe. It can be easily cultivated and produced in large quantities with currently available technology^21^. Indeed, a fungal-based genetic modification system has recently been employed that is relatively simple and easier than paratransgenic agents such as mosquito midgut bacteria and has already been used to control other vectors^13^. Because they are often free-living, environmentally friendly, highly effective, and can survive in the environment for months as spores^22^, fungi make excellent candidates for paratransgenesis. Once they enter the insect, they propagate in the insect’s body, and eventually, the fungal spores are released from the insect, completing their life cycle^13,23^.

It would be greatly worthwhile to obtain transgenic fungal strains that significantly reduce mosquito infectivity, as this could improve disease control without killing mosquitoes^24^. Efficient dissemination of such microorganisms is also a major challenge. In the current study of *A. oryzae*, we have shown that these fungi are almost perfect for this task. *A. oryzae* is readily transferred from larvae to adults. The fungi also stably colonize the mosquito's gut, where malaria parasites develop. We modified *A. oryzae* with a plasmid expressing a green fluorescent protein and molecules that selectively block parasite development in the vector. MP2 and EPIP are two identified peptides that inhibit *Plasmodium* development in the midgut of Anopheles^18,25–27^. Glucoamylase (GLA) protein is employed as a fusion protein to facilitate the secretion of GFP and peptides into the medium with increased efficiency. Two effector genes were introduced into pyrG auxotroph *A. oryzae* strain in two different constructs, *A. oryzae*-R_E_; GLA-GFP-EPIP (glucoamylase-green fluorescent protein-enolase-plasminogen interaction peptide fusion) and *A. oryzae*-R_M_: GLA-GFP-MP2 (glucoamylase-green fluorescent protein-midgut peptide 2 fusion).

EPIP is a peptide that prevents plasminogen from binding to the ookinete surface during parasite invasion of the midgut epithelium^28–30^, and MP2 binds to the surface of the mosquito midgut and inhibits *Plasmodium* invasion^28,31^. These effector proteins strongly inhibit *Plasmodium* by reducing oocyst formation^25,26,30,31^. The combination of highly effective *A. oryzae* as a carrier and EPIP and MP2 as effectors, therefore, appears promising for reducing the malaria infectivity to mosquitoes.

Here we present data from our study where transgenic *A. oryzae* were introduced into *An. stephensi* first-instar larvae (L_1_) via water for 24 h. Population dynamics of GFP-tagged fungi were determined by confocal microscopy and additionally confirmed by qPCR, western blotting, and culture. The ability of a microorganism to transfer trans-stadially in a vector population would depend on the ability of the microorganism to colonize the larval gut and transmit to adults over an extended period. Our findings suggest that *A. oryzae*-R is transmitted trans-stadially from larva to adult, making this fungus a suitable candidate for paratransgenesis. Recombinant fungal strains were tested for their ability to block *Plasmodium berghei* development in the *An. stephensi* mosquito. The results show that the recombinant *A. oryzae* can be used as a paratransgenesis system to inhibit oocyst formation in *An. stephensi* in the midgut by two anti-*Plasmodium* effector molecules, MP2 and EPIP, which may be promising for eradication of the parasite. This study highlights the possibility that *A. oryzae* may deliver different effector molecules against vector-borne diseases and for the purpose of disease control under natural conditions. Optimization of this method will help us develop new strategies to treat vector-borne diseases in the future.

## Materials and methods

### Ethics statement

All procedures were performed in accordance with the terms of local animal welfare laws, guidelines, and policies of Bezmialem Vakıf University, Beykoz Institute of Life Sciences and Biotechnology and the animal protocol was approved by the Animal Care and Use Committee of Bezmialem Vakıf University (protocol number 2021/155) and the genetic modification experiments have been approved by Bezmialem Vakif University and TUBITAK (The Scientific and Technological Research Council of Turkey).

### Mosquito rearing and maintenance

*An. stephensi* (WT strain) were reared under standard insectary conditions in climate-controlled rooms, maintained at 27 °C (± 1), 80% relative humidity, and a 12 h:12 h light: dark photoperiod. Larvae were fed on Tetramin® ground fish food (Tetra, Melle, Germany), using 0.1 mg/larva for the first larval stages and 0.3 mg/larva for the other three larval stages in rearing trays of 10 × 25 × 8 cm filled with 1 L of water at a density of approximately 0.3 larvae/cm^2^. Pupae were collected and placed into holding cages and provided with an ad libitum supply of 10% sucrose solution for adult mosquitoes^32^.

### Generation of *A. oryzae* expressing two antimalarial peptides and GFP

**Figure 1:**
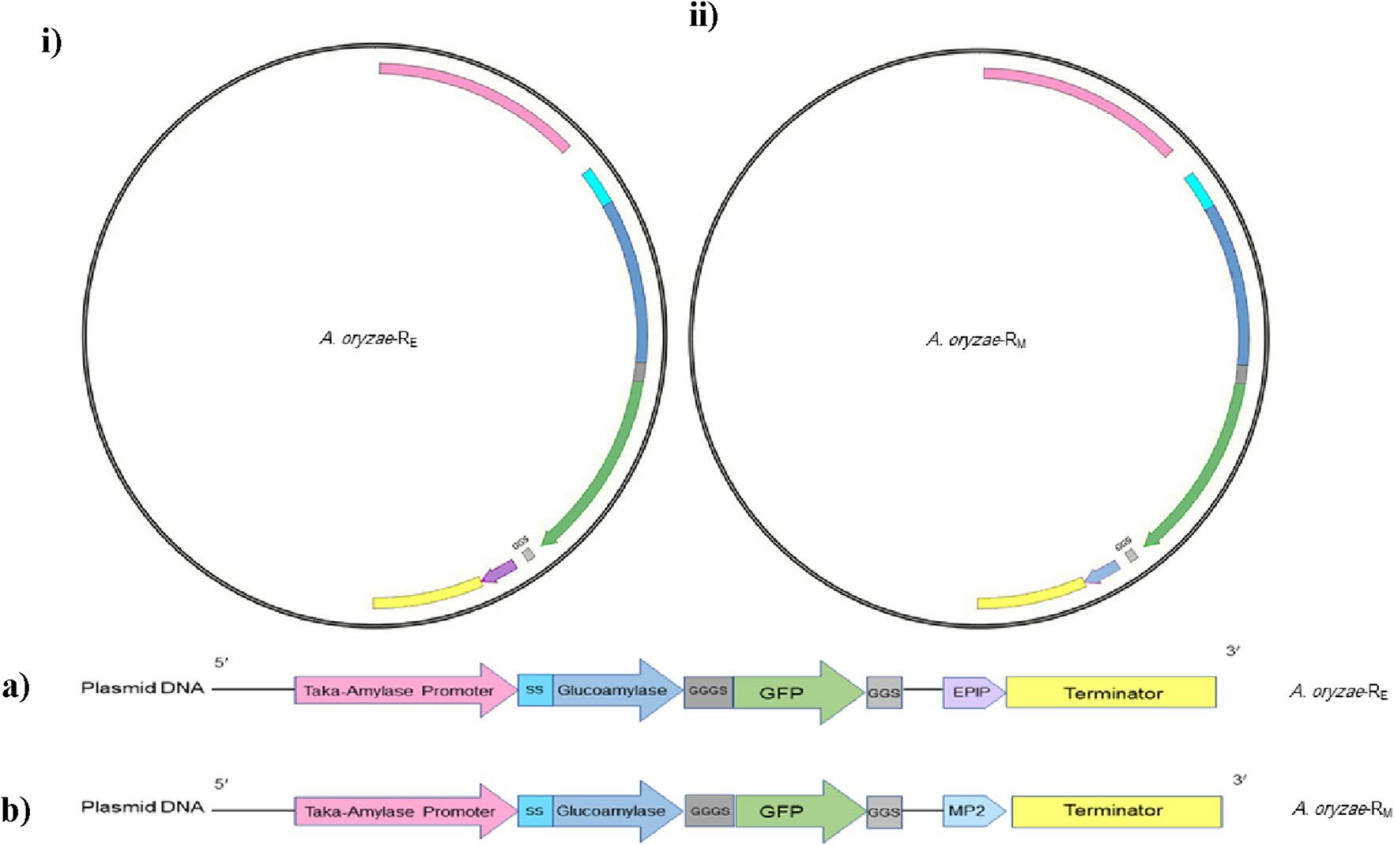
Expression vector. (i) Expression vector of *A. oryzae*-R_E_ and (ii) *A. oryzae*-R_M_, (a) Linear plasmid of *A. oryzae*-R_E_ containing the Taka amylase promoter, glucoamylase-green fluorescent protein-plasmodium enolase-plasminogen interaction peptide fusion and terminator. (b) Linear plasmid of *A. oryzae*-R_M_ containing the Taka amylase promoter glucoamylase-green fluorescent protein-midgut peptide 2 fusion and terminator.

*A. oryzae* strain RIB40 (cat no: 42149) was purchased from ATCC (American Type Culture Collection). A pyrG (-) *A. oryzae* RIB40 strain which was generated in our laboratory for an earlier study was used for fungal transformation^33^. Escherichia coli Mach TM was employed for DNA cloning, plasmid amplification. The recombinant *A. oryzae* was modified to express and secrete GFP and antimalarial effector proteins MP2 and EPIP, using two different constructs separately, *A. oryzae*-RE: GLA-GFP-EPIP (glucoamylase-green fluorescent protein-*plasmodium* enolase-plasminogen interaction peptide fusion) and *A. oryzae*-RM: GLA-GFP-MP2 (glucoamylase-green fluorescent protein-midgut peptide 2 fusion). All genes were cloned into a customized expression vector that drives expression using TAKA amylase promoter (Fig. 1). Genetically modified fungi were cultured on Czapek-Dox (CD) medium plates at 30 °C for 4–6 days after incubation at 30 °C. Individual distinct fungal colonies were isolated from the plate, the spore layer was carefully harvested and inoculated into dextrin-peptone-yeast (DPY) liquid medium and incubated at 30 °C, 180 rpm for 5–7 days. The presence of the *A. oryzae*-R was verified by confocal microscopy.

### Confirmation of trans-stadial transmission detection of *A. oryzae*-R in *An. stephensi* using confocal microscopy

To investigate the colonization and adaptation of *A. oryzae*-R in the gut of *An. stephensi* larvae and transfer to the midgut of adult mosquitoes, approximately 4 × 10^6^ conidia/mL were inoculated into 100 mL of DPY medium and then incubated at 30 °C for 5–7 days for GFP expression, following incubation, the fungi were verified by confocal microscopy to confirm the presence of the recombinant fungi by GFP detection. *A. oryzae*-R (*A. oryzae*-R_E_ and *A. oryzae*-R_M_ mixed in equal ratios) were added to water containing larvae of the first instar (L_1_) of *An. stephensi* at a final concentration of 1:200 and incubated for 24 h^34^.

The submerged larvae were collected after 24 h, washed twice in separate baths with distilled water to remove fungi on the body surface of the larvae, and were then transferred to fresh water to avoid contamination with external fungi. The presence of the modified fungi was monitored at four time points (days 1, 4, 7, and 10) after inoculation with the fungi and again at four time points (days 1, 4, 7, and 10) after hatching of the adults. Individual larvae and adult mosquitoes were surface sterilized by washing in 75% ethanol and then rinsing in sterile PBS prior to dissection.

At least 5 larvae from each time point were collected, dissected, and examined using a confocal microscope (Leica DMi8 laser scanning confocal microscope, 20 × objectives) for the presence of GFP to assess fungal viability and activity in the larval gut. To investigate *A. oryzae*-R colonization in the larval gut, resistance in the gut environment, and transmission to the adult mosquito midgut (trans-stadial transmission), at least 5 female mosquitoes were collected, surface sterilized, and dissected to obtain their midguts at specific time points, and then examined for the presence of GFP-expressing fungi using confocal microscopy. In addition, *An. stephensi* larvae were reared in trays with no *A. oryzae*-R as a negative control.

### Detection of GFP-tagged *A. oryzae* from *An. stephensi* by quantitative polymerase chain reaction (qPCR)

Larvae were sampled at four time points: 1-, 4-, 7-, and 10-days post inoculation in the larval and at the same time points (days 1, 4, 7, and 10) after emergence as adults. The specimens were surface sterilized with 75% ethanol and rinsed in sterile PBS^35^. Genomic DNA was extracted from experimental and control groups using a DNeasy® Blood & Tissue Kit (QIAGEN) according to the manufacturer’s instructions. Groups of 5–10 larvae and 5–10 adult mosquitoes were collected separately at each time point. The assay allowed specific detection of a 128 bp sequence of the pyrG gene in *A. oryzae*. The sequences of the forward and reverse primers were 5′ATATCCTCTCCGATTTCAGCGAAGA and 5′ATGGTACTGCTTTTGGACTGTGTTT, respectively. qPCR was performed using SYBR Green Master Mix (Bio-Rad, USA). The 18S ribosomal RNA gene was used as a reference gene for normalization. Reactions were performed in a final reaction volume of 20 μl with 200 nM forward and reverse primers and 50 ng/μl of the DNA template^36–38^. The assay was performed on a qPCR instrument Rotor-Gene Q System (QIAGEN), and data were acquired in real time. Three experimental replicates were used for qPCR. The optimized cycling conditions consisted of 94 °C for 4 min, followed by 40 cycles of 95 °C for 10 s, 60 °C for 15 s, 72 °C for 10 s^38^. Melting curve analyses were performed to check for primer–dimer formation. CT values were determined for all PCR reactions, and a comparative CT method was used to calculate the copy number of the expressed pyrG gene. The relative quantity of expressed gene was calculated as RQ = 2^−(∆∆CT)^^39,40^.

### Western blot evaluation of secreted GFP by *A. oryzae* isolated from the larvae and the adult mosquito guts

To determine the expression and secretion of GFP fusion protein levels by recombinant *A. oryzae* in the larvae and the adult mosquito’s midguts, groups of 20–50 larvae and adult mosquitoes were examined at two time points (days 1 and 10) after inoculation at the larval stage and post emergence at the adult stage on days 1 and 10. Prior to dissection, individual larvae and adult mosquitoes were surface sterilized by washing in 75% ethanol and then rinsing in sterile PBS. The larvae and adult cohort that had been inoculated with *A. oryzae* wild type served as negative controls. Protein isolation from the dissected guts in larval and adult stages was performed according to the method of^41^. Western blotting was performed as previously described^42–44^. Protein samples were separated using 8% SDS-PAGE and transferred to a nitrocellulose membrane (Amersham Protran Premium 0.45 μm NC, GE Healthcare). The membrane was incubated in blocking buffer (0.1% Tween-20 with 5% w/v nonfat dried milk) and probed with GFP monoclonal antibody (GF28R), HRP (Invitrogen). Supernatant from cultures of *A. oryzae* producing GFP fusion protein was used as a positive control. Fungal cultures were centrifuged at 4000 × g for 15 min at 4 °C, and an equal volume of the culture supernatant was concentrated using Amicon Ultra-15 centrifugal filter units.

### Isolation and characterization of *A. oryzae*-R from *An. stephensi* larvae and adult mosquito’s midguts by culturing

The specimens were selected at four time points (days 1, 4, 7, and 10) after larval stage inoculation and days 1, 4, 7, and 10 after adult hatching. Untreated larvae and adults were used as negative controls. Larval and adult mosquito surfaces were sterilized before dissection with 75% ethanol, and dissected guts were individually homogenized in 100 μl sterile 1% PBS (10 samples at each time point in each stage). Homogenates were plated on Czapek-Dox (CD) medium plates containing 50 μg/ml kanamycin and 100 μg/ml ampicillin and incubated at 30 °C for 3 days. Single distinct fungal colonies were isolated from each plate and inoculated into dextrin-peptone-yeast (DPY) liquid medium and then incubated for 5–7 days at 30 °C for GFP expression^45^. Thereafter, the presence of GFP was observed under the confocal microscope.

### The effect of *A. oryzae*-R on longevity and blood feeding behavior of *An. stephensi*

*A. oryzae*-R was introduced to the larvae via water. The 2-day-old larvae were incubated for 24 h. A control group of larvae were reared in a tray without fungi. Survival of the larvae and emerged adults were monitored daily. A total of 150 larvae and adult mosquitoes per group were studied separately (50 per group × 3 replicates = 150). Five days after emergence, adult female mosquitoes were starved for 8 h and then allowed to feed on a blood meal for 1 h. To calculate the success of the blood meal, the number of blood-fed and non-fed females in the two groups were counted and the blood feeding proportions of the two groups were statistically compared using a t-test of two proportions. A total of 150 adult mosquitoes were studied per group (50 mosquitoes per group × 3 replicates = 150 mosquitoes)^25^.

### The effect of antimalarial effectors expressed by *A. oryzae*-R on mosquito fecundity and fertility

Wild-type and recombinant *A. oryzae* were introduced into 2-day-old larvae via water for 24 h. Fungal minus groups serve as control groups. For fecundity and fertility assays, a method was applied to stimulate females to lay eggs as previously described^46^. Blood-fed females were fed with 10% sucrose solution and held at 27 °C (± 1) and 80% relative humidity for 3 days to fully reach the gravid stage. The female mosquitoes were then carefully placed individually in 50 ml conical tubes with a moistened filter paper at the bottom of the tube. The tubes were covered with cotton. Females that had laid eggs were carefully removed from the tubes. The number of laid eggs and hatched eggs were counted. The fecundity of the fed female mosquitoes was calculated as the number of eggs laid per female. A total of 90 adult mosquitoes were studied per treatment (30 mosquitoes per each group × 3 replicates = 90 mosquitoes). Fertility (proportion of larvae that hatched from the eggs) was calculated as the percentage of larvae that emerged after hatching^46^.

### *Plasmodium berghei* infections

*P. berghei* strain ANKA (MRA-868, MR4, ATCC, Manassas, Virginia) was maintained by cyclic passage through BALB/c mice and *An. stephensi* mosquitoes following a standard protocol. Mice were anesthetized with Rompun-Vetalar (3:1.5 mg/kg; Bayer and Parke-Davis Veterinary), then wild-type *An. stephensi* were fed on anesthetized mice. Infectivity of each mouse was determined by measuring parasitemia in Giemsa-stained tail-blood smears. Those mice with 1–2 exflagellations/field under a 20 × objective were used for mosquito infections. Blood-fed mosquitoes were maintained at 21 °C (± 1) and 70% relative humidity.

### *Plasmodium berghei* transmission blocking assay

Approximately 4 × 10^6^ conidia/ml as a starting point were inoculated into 100 ml of DPY medium and then incubated for 5–7 days at 30 °C for GFP expression. After incubation, the fungi were verified by confocal microscopy to confirm the presence of GFP. The recombinant and wild-type fungi were separately further diluted 1:200 in water and first instar (L_1_) larvae of *An. stephensi* were incubated for 24 h. The L_1_ were collected after 24 h, rinsed three times in sterile PBS to remove fungi from the larval body surface, and then transferred to distilled water. Different conditions were prepared to expose the larvae: 1—no fungi (negative control), 2—wild-type *A. oryzae*, 3—*A. oryzae*-R_E_, 4—*A. oryzae*-R_M_, and. 5—*A. oryzae*-R (*A. oryzae*-R_E_ + *A. oryzae*-R_M_). After hatching to adults, mosquitoes were fed a blood meal containing *P. berghei* ANKA gametocytes for 30 min on female 8-week-old BALB/c mice. Fully fed females were sorted and maintained in cages at 21 ± 1 °C, 70% relative humidity, with 10% sucrose solution available. The effect of parasite development was determined in 10–12 days post-infection by counting the number of oocysts in the midgut using confocal microscopy. A total of 90 adult mosquitoes were examined per treatment (30 per treatment × 3 replicates = 90).

### Institutional review board statement

The study was conducted in accordance with the terms of local animal welfare laws, guidelines, and policies of Bezmialem Vakıf University, Beykoz Institute of Life Sciences and Biotechnology, and the animal protocol was approved by the Animal Care and Use Committee of Bezmialem Vakıf University (protocol number 2021/155).

## Results

**Figure 2:**
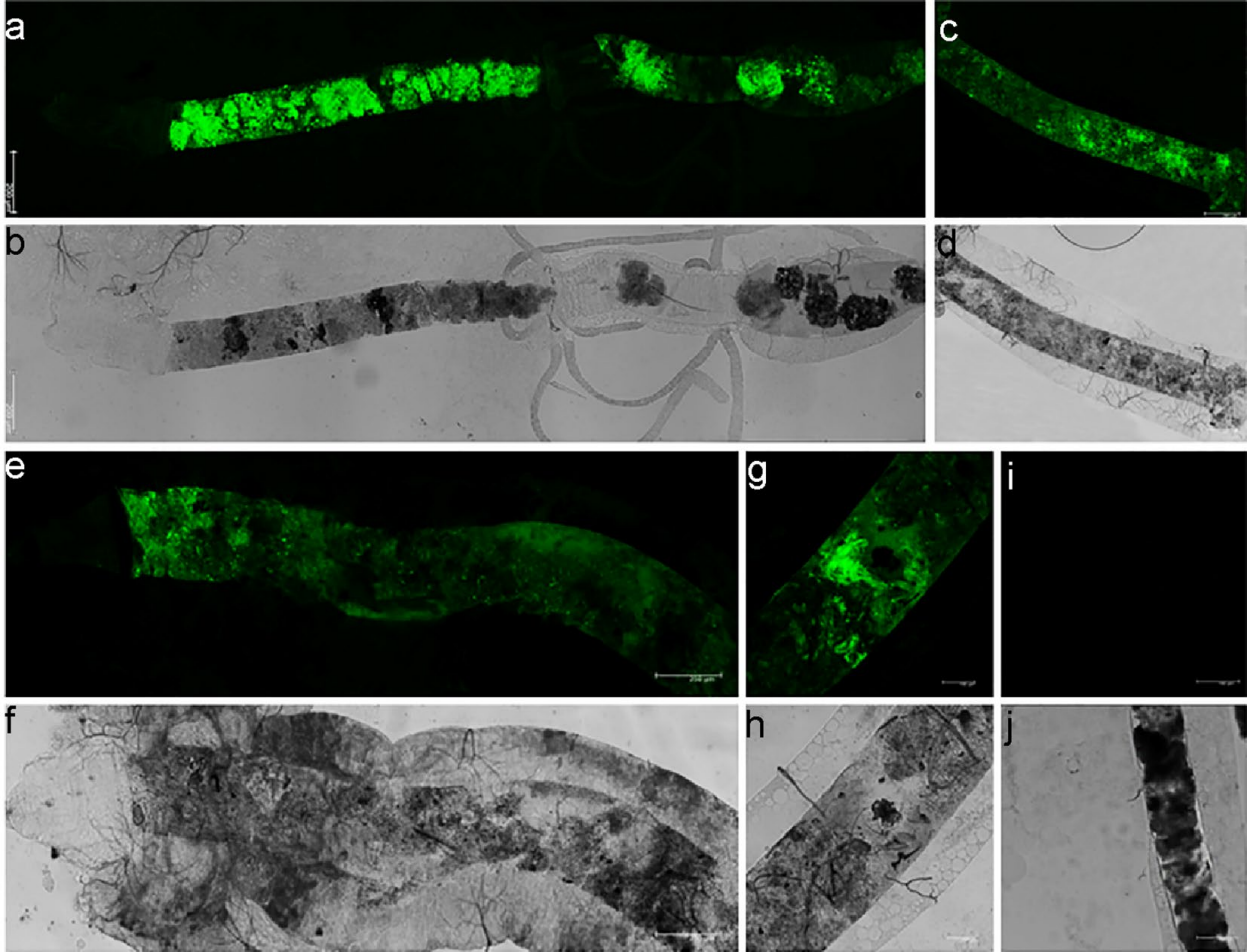
Colonization of *A. oryzae*-R at different time points in the gut of *An. stephensi* larvae, detected by expression of the GFP gene in colonized fungi. The population dynamics of *A. oryzae*—R were visualized by confocal microscopy. (**i**, **j**) negative control, (**a**, **b**) the gut of L_1_ *An. stephensi* larva day 1 (**c**–**h**) 4,7 and 10 The experiments were repeated three times with similar results.

**Figure 3:**
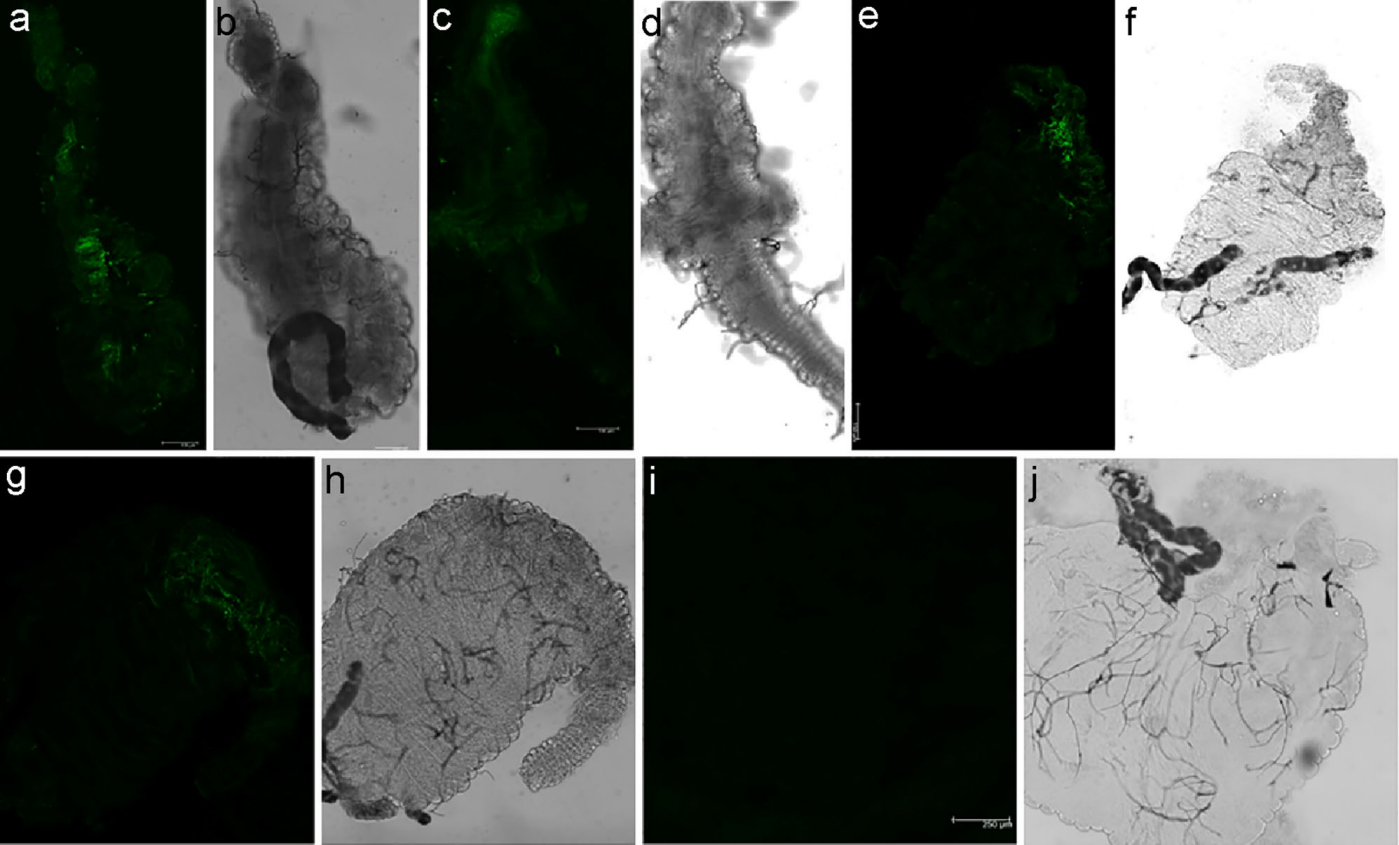
Colonization of *A. oryzae*-R in the adult mosquito midgut at different time points shown by GFP expressed in the transgenic fungi. (**a**, **b**) adult *An. stephensi* midgut day 1 (**c**–**h**) 4, 7, and 10, respectively, (**i**, **j**) negative control. The experiments were repeated three times with similar results.

*A. oryzae* was genetically engineered to secrete the anti-*Plasmodium* effector proteins EPIP and MP2. The aim of the present study was to investigate the possible ability of the transgenic fungi to colonize and adapt in the larval gut of *An. stephensi* and transfer to the adult as a paratransgenesis model, as well as the possibility of inhibiting the development of *P. berghei* in the mosquito gut. If they persist consistently within the larval gut, we wanted to know whether the fungi can transfer to the adult mosquito’s midgut (trans-stadial transmission). The persistence of the *A. oryzae*-R in the midgut of *An. stephensi* was monitored to investigate the dynamics and maintenance of these fungi during the transition from larva to adult. To measure *A. oryzae* colonization and persistence in the larval gut, a fluorescent protein gene coding for an enhanced green fluorescent protein (GFP) was incorporated into the genome of *A. oryzae*. To evaluate these parameters, GFP-labelled fungi were inoculated via water into *An. stephensi* first-instar larvae (L_1_) for 24 h. In the environment where *Anopheles* mosquitoes naturally live, larvae and mosquitoes are not sterile. Thus, *A. oryzae* would also have to compete with other species in the microbiota that could affect these parameters. However, using laboratory colony mosquitoes limited any potential interference from the wide array of microbiota found in wild breeding sites that may affect our data. The colonization of *A. oryzae*-R in the larval gut was assayed at four time points. At both stages, the midgut was dissected and analyzed for the presence of GFP-marked fungi in the midgut by confocal microscopy. During mosquito metamorphosis, the gut microbiota is eliminated at the larval stage^47^, but *A. oryzae*-R remained in the midgut of the adults that emerged from these larvae (Figs. 2, 3). A high concentration of GFP expressing fungi was observed on the first day of the larval stage, numerous fungi were active and occupied most of the gut lumen, including the anterior midgut (AMG) and the posterior midgut (PMG), some autofluorescence is seen in the hindgut region (HG). This indicates that fungal entry occurs during ingestion. On day 4, the intensity of GFP was relatively high and plenty of the transgenic fungi were observed. However, on day 7, they decreased relatively, though many fungal cells were still active and expressing GFP in the gut. The presence of GFP was confirmed on day 10, but it was lower than on the previous days (Fig. 2). We assume that the non-fluorescent fungi may be inactive, dead, or excreted through the feces. GFP expression was relatively low in the adult stage compared to the larval stage. Despite a low number of transgenic fungi in the midgut of the adult mosquitoes, the level of green fluorescent protein was remarkable and consistent in the midgut of adult mosquitoes throughout the study period (Fig. 3).

The fungi colonized *An. stephensi* very well at different developmental stages, including larval instars 1–4 (L_1_–L_4_) and adult mosquitoes. Transgenic *A. oryzae* were transmitted trans-stadially in *An. stephensi* despite several molting events during larval stages as well as hydrolytic processes during metamorphosis. A short contact time (24 h) between the fungi and L_1_ larvae was sufficient for the larvae to acquire the fungi from the water of the larval habitat. Examination of the untreated larvae and the adult mosquitoes by confocal microscopy confirmed the absence of transgenic fungi in the negative controls.

**Figure 4:**
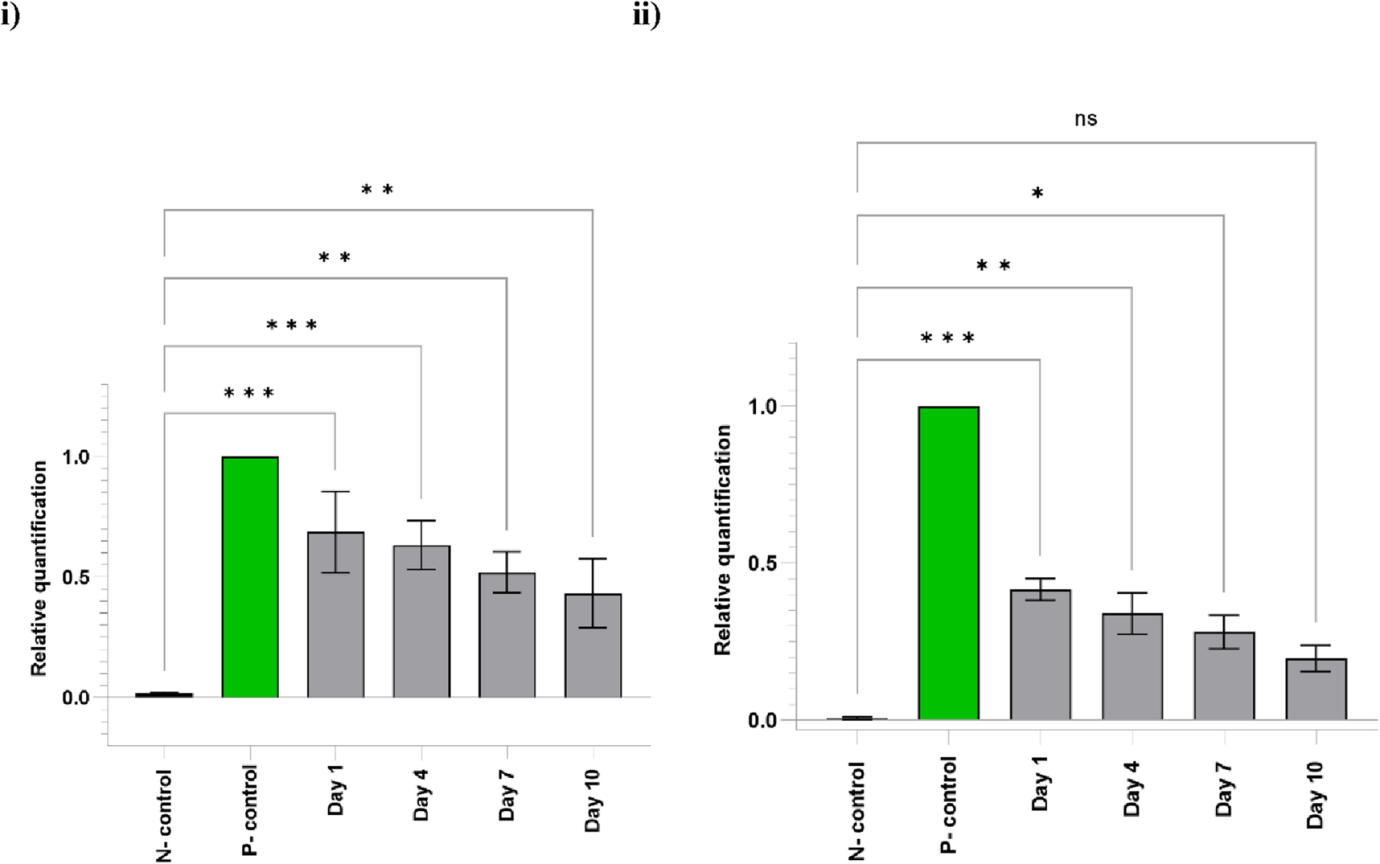
Relative quantification of pyrG gene amplification by real-time quantitative PCR. (i) larvae; (ii) adult mosquito midguts at four-time points (days 1, 4, 7, and 10), negative control (N-control), positive control (P-control) (*p* < 0.05). Data were pooled from three replicates. The expected ∆∆CT ratio = 1 when the pyrG gene is expressed.

To quantify the copy numbers of the recombinant fungi, quantitative PCR was used. Larvae and adult mosquitoes were examined at the intervals mentioned above. The size and quality of the amplified qPCR products showed clear specific bands on agarose gel for both the larva and adult mosquito samples (data not shown). The qPCR amplification of the specimens showed the presence of the fungi in the gut of the larvae and adult mosquitoes. Quantification was done by targeting a 128 bp sequence of the *pyrG* gene (Fig. 4). The means of amplification efficiencies per amplicon in the larval samples were 69% (day 1), 64% (day 4), 52% (day 7), and 43% (day 10). It decreased at day 10 compared to day 1. qPCR analysis at the larval stage between the negative control and days 1, 4 and 7 showed highly significant differences, *p* < 0.05 (Fig. 4i). For the adult mosquitoes, the amplification efficiencies were 41.6%, 34.0%, 28.0%, and 19.6% on days 1, 4, 7, and 10, respectively (Fig. 4ii). Comparison between the negative control and days 1, 4 and 7 was significant in the adult specimens. The difference between the negative control and day 10 was not significant (*p* = 0.082). Therefore, by the qPCR results, confirmed the trans-stadial transmission of the transgenic *A. oryzae*. Data demonstrated a mass of fungi in the larval gut on day 1 (69%), which decreased to 20% in the adult mosquito’s midguts on day 10.

**Figure 5:**
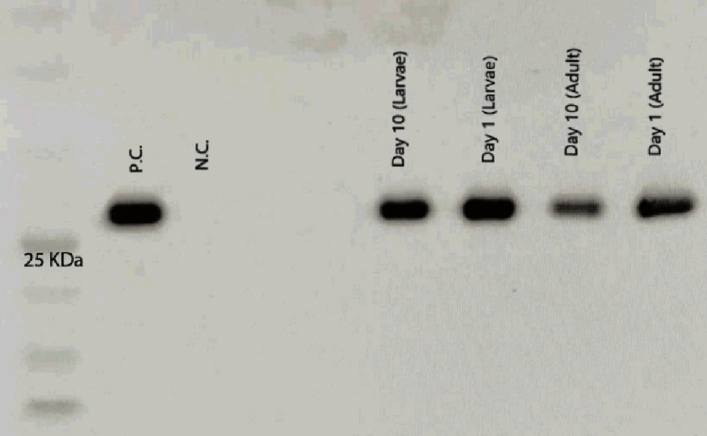
Western blot analysis of protein secretion. Aliquots of  equal protein concentration were analyzed by Western blot using the GFP monoclonal antibody. *A. oryzae*-R was inoculated into *An. stephensi* L_1_ larvae via water for 24 h, except for the negative control, which was inoculated with wild-type *A. oryzae* (N.C.). The concentrated supernatant of *A. oryzae*-R served as the positive control (P.C.), on the first and tenth day at the larval stage and on day one and day ten at the adult stage. Shown are the bands for the cleaved forms of GFP (28 kDa).

We used western blot to validate the expression and secretion of the fusion protein (GFP) by recombinant fungi. GFP levels at two-time points probed in larvae and adults are shown in Fig. 5. All samples showed presence of secreted GFP.

**Figure 6:**
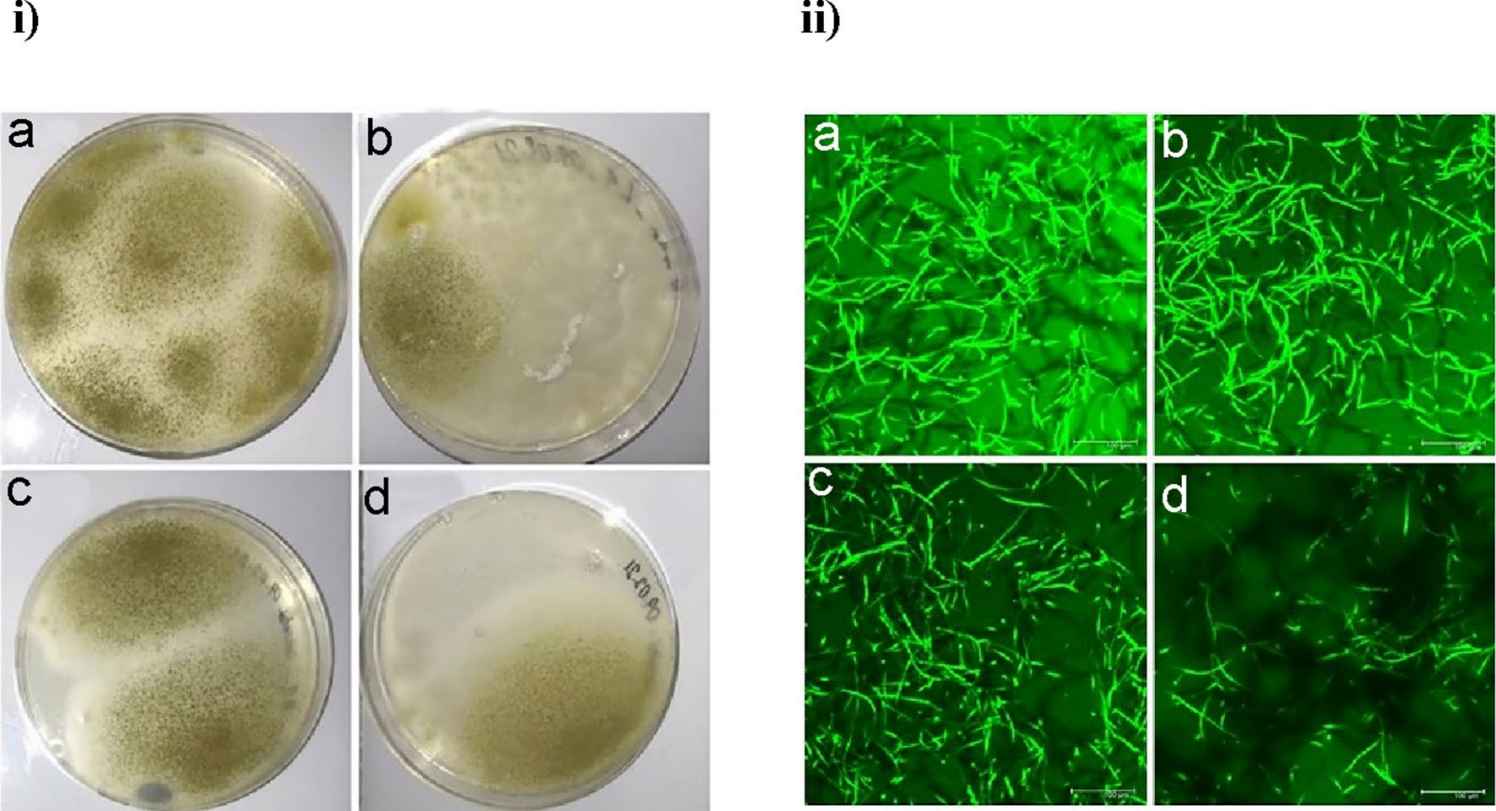
The isolates with GFP expression were visualized by confocal microscopy. *A. oryzae*-R was introduced into *An. stephensi* first instar. The larva and the adult mosquito’s midguts were inoculated on CD agar plates containing ampicillin and kanamycin separately. (i) day 1 (a) and 10 (b) in larval stage, c and d day 1 and 10 of adult stage. *A. oryzae*-R isolated from the larvae and the adult mosquito midgut (ii), (a, b) day first and tenth of larval stage, (c, d) day first and tenth of adult stage. Scale bar, 100 mm. (The experiments were repeated three times with similar results).

In order to determine the presence of viable *A. oryzae*-R colonization of larvae and emerged adults, midgut homogenates were plated onto CD/kanamycin, ampicillin agar plates (Fig. 6i), followed by checking for fluorescent colonies. Some colonies on the CD agar plates were expected to represent the transgenic fungi population associated with both the larvae and adult mosquito midguts. The isolates were visualized by confocal microscopy after incubation (Fig. 6ii). Confocal microscopy results showed GFP expression in all samples at both stages, evidence for the ability of the fungus to transmit trans-stadially in *An. stephensi* mosquitoes. These assays provide baseline information on the adaptation of these fungi to the gut environment of larval and adult mosquitoes.

**Figure 7:**
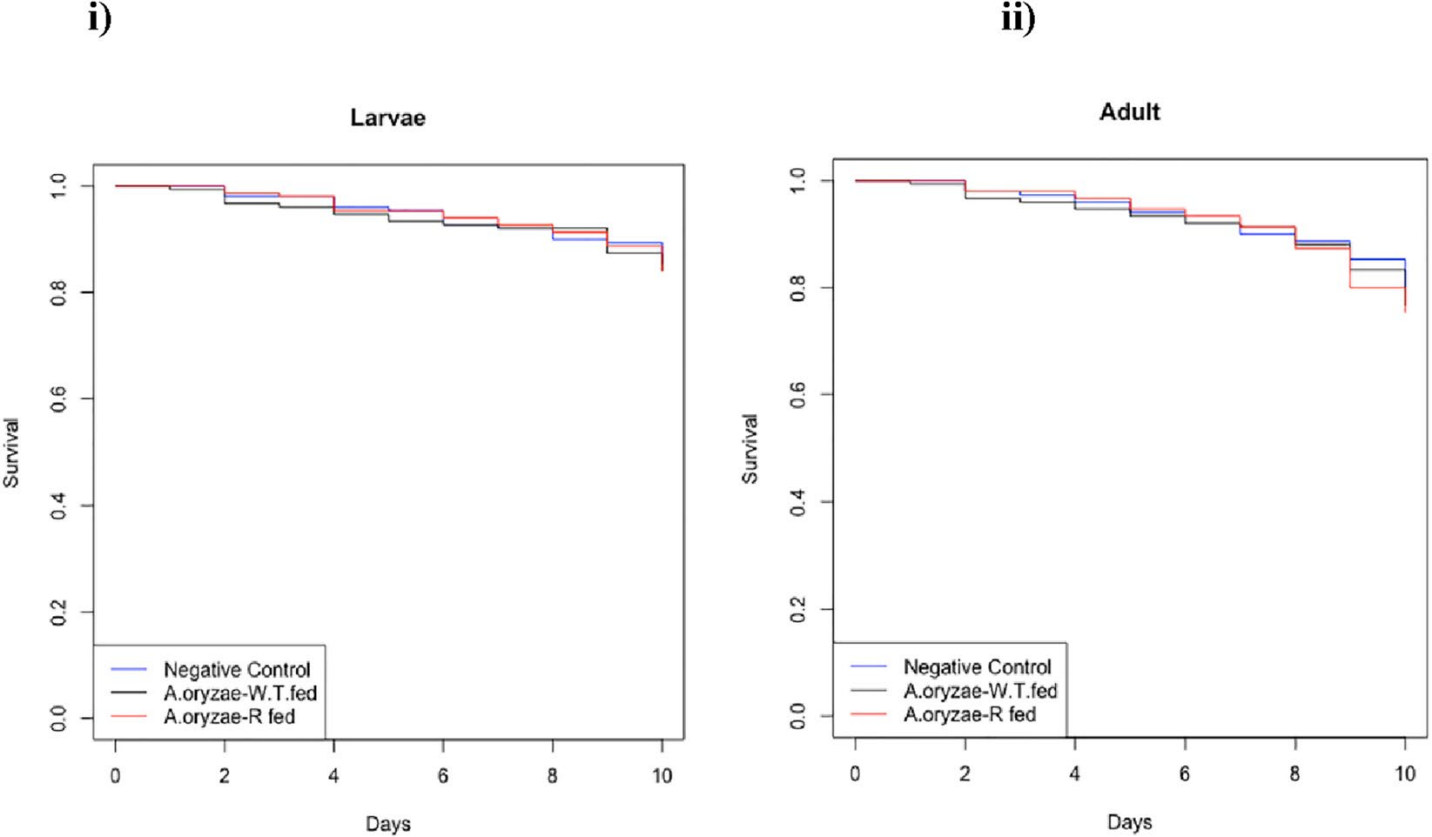
Effect of *A. oryzae*-R on larval and adult mosquito survival. (i) Survival rates of *An. stephensi* larvae after feeding on *A. oryzae* (ii) Survival rates of mixed male and female *An. stephensi* adult mosquitoes after emergence. Differences in larval and adult mosquito survival rates between the *A. oryzae*-fed and negative control groups were analyzed using Cox Regression (Cox, 1972) (*p* < 0.05) in R software version 4.0.3. Cox Regression compared survival of the different groups and revealed nonsignificant differences in overall survival rates at both larval and adult stages. Larvae and adult survival did not differ significantly between the *A. oryzae*-fed and negative control groups (larvae: logrank test, res-cox, df = 1, *p* = 0.8) and (adults: logrank test, res-cox, df = 1, *p* = 0.3). Data are from three biological replicates with 50 mosquitoes per group.

We next wanted to rule out any effects of recombinant fungi themselves on the viability or development of the mosquitoes, and the success of the paratransgenesis strategy with minimal fitness costs to survive in the gut of larval and adult mosquitoes in a competitive environment. Two-day-old larvae of *An. stephensi* (L_1_) were separately inoculated with the wild type *A. oryzae* and *A. oryzae*-R. A subset of *Aspergillus*-free mosquitoes was tested for possible contamination as a negative control group. Survival of larvae and adult mosquitoes carrying or not carrying *A. oryzae* was compared (Fig. 7i, ii). There was no significant difference in the survival rate between the groups in the larval and adult stage which is an indication that the fungal infection did not affect the larvae and the mosquito’s developmental rate.

**Figure 8:**
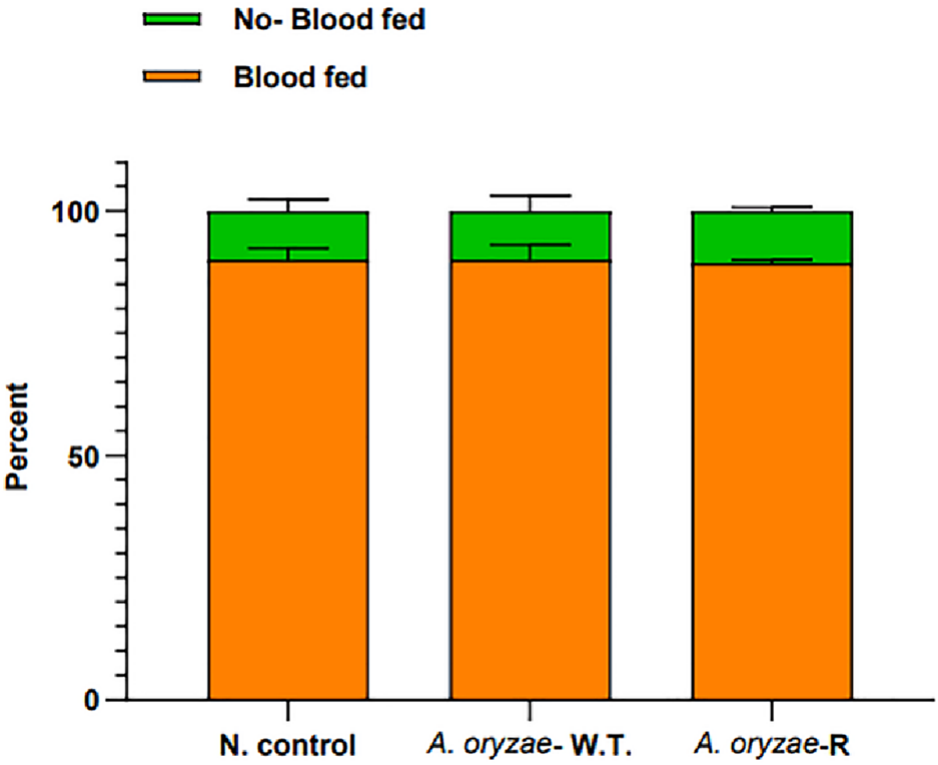
Effect of recombinant *A. oryzae* on adult mosquito blood feeding behavior. Recombinant *A. oryzae* does not impact mosquito blood feeding behavior. Percentages are means of three biological replicates of 50 mosquitoes each (mean ± SEM). There is no significant difference between the groups treated with the *A. oryzae* infected (recombinant and wild type) and the negative control (Tukey’s multiple comparisons test was used to test significance).

**Figure 9:**
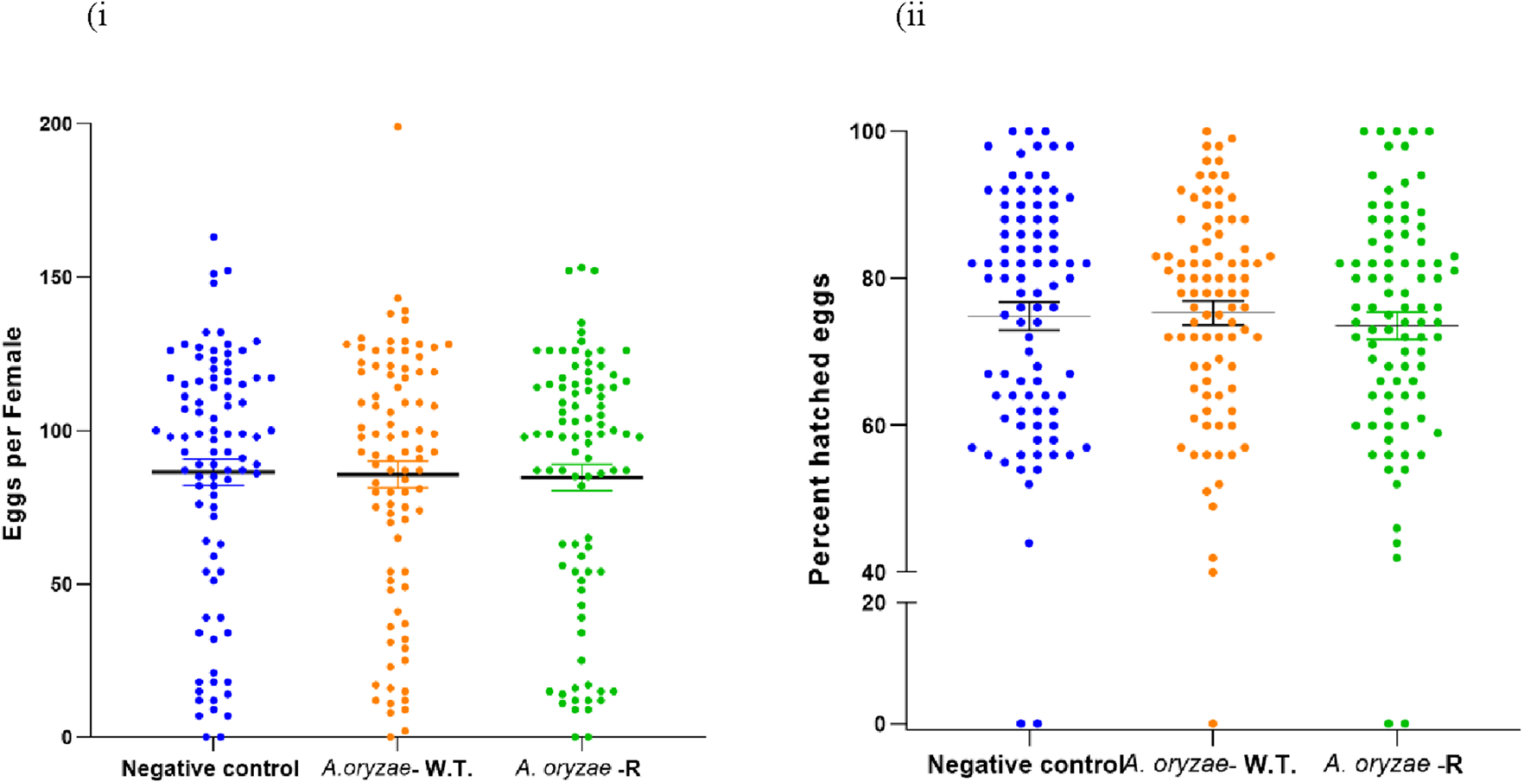
Impact of antimalarial effectors expressed from recombinant *A. oryzae* on mosquito fecundity and fertility. (i) Number of eggs produced per female (mean ± SEM). There was no significant difference between the groups. (ii) Percentage eggs hatched (mean ± SEM). Data were compared within treatments using Kruskal–Wallis test and pooled based on the absence of any significant differences from three independent experiments. Comparisons between samples were then analyzed using the Kruskal–Wallis test, and statistical comparisons of the number of eggs produced per female and the number of hatched eggs with those of negative controls were analyzed separately using the Mann–Whitney test.

In order to rule out any effects of transgenic *A. oryzae* on fecundity or fertility of the mosquitoes, blood feeding of mosquitoes incubated with or without *A. oryzae* (recombinant and wild type) was compared. There was no effect on blood-feeding behavior in either group (Fig. 8). On average, blood-feeding success was 89.3, 90, and 90% for the *A. oryzae* infected (recombinant and wild type) and uninfected mosquito groups, respectively. All females were successfully fed with no interruptions or partial feedings. There was no evidence of reduced blood intake in *Aspergillus*-infected or non-infected mosquitoes. Mosquitoes incubated with or without *A. oryzae* were fed blood and the number of eggs laid per female (fecundity) and the percentage of eggs hatched (fertility) were evaluated. The number of eggs laid by the negative control group, which was not incubated with the fungi, was on average similar to those laid by the fungus-fed mosquitoes (recombinant fungi and wild type). The mean value of eggs laid by control mosquitoes was 86.4 per female, whereas this value was 85.6 (wild-type) and 84.7 (*A. oryzae*-R) eggs per female for mosquitoes incubated with *A. oryzae*. There was no significant difference in the fecundity of female mosquitoes between the three groups (*p* = 0.98) (Fig. 9i). The fertility of mosquitoes harboring *A. oryzae* and the control group was not significantly different (*p* = 0.72) (Fig. 9ii). The mean percentage of viable eggs incubated by *A. oryzae* was 75.3% (wild-type) and 73.5% (*A. oryzae*-R), whereas the percentage of viable eggs laid by control females was 75% in their first gonotrophic cycle. These results indicate that feeding of transgenic *A. oryzae* to *An. stephensi* mosquitoes does not alter their biology in general and makes their use as a carrier of molecules targeting pathogens as a viable solution.

**Figure 10:**
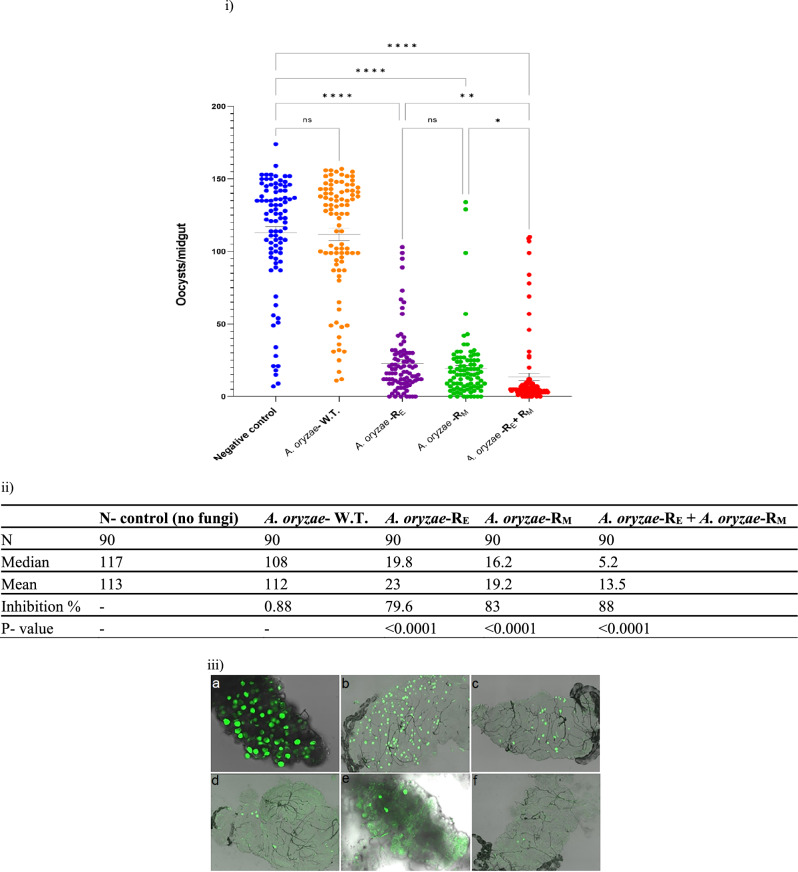
Inhibition of *P. berghei* infection of *An. stephensi* by recombinant *A. oryzae* modified to secrete anti-*Plasmodium* effector molecules (EPIP and MP2). (i) Circles represent the number of oocysts per individual midgut, and the horizontal lines show the median of oocysts per midgut (also shown in the table). Data within treatments from three biological replicates were not significantly different (Kruskal–Wallis test) and were subsequently pooled. Comparisons between treatments for the pooled data were analyzed also by the Kruskal–Wallis test, medians were compared using Dunn’s test, and statistical comparisons of oocyst numbers with those of negative controls were analyzed using the Mann–Whitney test. A value of *p* < 0.05 was considered to be statistically significant. (ii) Summary data from (i) N, number of mosquitoes analyzed; Median, median number of oocysts per midgut; Mean, mean number of oocysts per midgut; inhibition %, percent inhibition of oocyst formation compared to control without fungi. (iii) GFP-expressing *P. berghei* oocysts 10 days post-infection of mosquitoes. Oocyst numbers in the midguts were counted by microscopy (40 × objective). (a) Negative control (no fungi), (b) wild-type *A. oryzae*, (c) *A. oryzae*-R_E_, (d) *A. oryzae*-R_M_, (e, f) *A. oryzae*-R_E_ + *A. oryzae*-R_M_ (arrows point to the oocysts).

To determine whether the recombinant *A. oryzae* effectively impairs the development of oocysts in the midgut, mosquitoes carrying recombinant fungi were fed on mice infected with *P. berghei*, whereupon the number of oocysts was determined at day 10–12 post-infection (Fig. 10). Compared to the control groups, the introduction of *A. oryzae*-R dramatically blocked oocyst development (79.6–88.5%), which is consistent with the observations of Pumpuni et al. and Yoshida et al.^48,49^. The recombinant *A. oryzae* expressing MP2 had the lowest infection rate (83%) and significantly lower mean oocyst intensity (19.2 oocysts/midgut). An equal mixture of fungi expressing EPIP reduced mean oocyst intensity by 79.6% (23 oocysts/midgut), whereas a mixture of fungi expressing MP2 + EPIP reduced mean oocyst intensity by 88.5% (13.5 oocysts/midgut) (Fig. 10), despite relatively high infection rates.

## Discussion

The first step for paratransgenesis when controlling malaria or other vector-borne diseases is identification of an adaptable symbiont. It is a complex process and proper studies should be performed to understand the relationship between vectors and their microbiota^22,26^. Our results demonstrate that the larvae of *An. stephensi* can obtain *A. oryzae*-R from the water in which they are reared. Recombinant fungi were isolated in high numbers from the larval gut the day after inoculation of the water with fungal spores. The data are consistent with those of Lindh et al.^50^ who suggested the possibility of introducing transgenic microorganisms into a mosquito population in the field through breeding sites and sugar-baits. It might be easier and more practical to introduce genetically engineered microorganisms into larval stages than to adult mosquito population. The larval stages of mosquitoes develop in water and feed on organic material such as microorganisms and particles from their environment so any microorganisms in the water can be ingested by the larvae^51–54^. This behavior makes it relatively easier to expose larvae to target microorganisms. Consequently, the larvae host a varied and mutable microbial community^53,55,56^, any of which could be considered as valid targets for paratransgenesis.

The experimental results presented here show the colonization pattern and trans-stadial transmission of *A. oryzae*-R from the larvae to the adult mosquitoes of *An. stephensi*. In different groups of mosquitoes, trans-stadial transmission of microorganisms from the larval to the adult stages includes bacteria in *Anopheles* mosquitoes^26,53,57–64^, and dengue virus in *Aedes aegypti*^65^, La Crosse virus in *Ochlerotatus triseriatus*^66^, Rift Valley fever virus in *Culex pipiens* and *Ae. circumluteolus*^67^, *Francisella tularensis* bacteria in *Ae. sticticus, Ae. vexans, and Ae. punctor*^68^, and *CuLCrVvirus* in *Bemisia tabaci*^69^. In the present investigation, the transgenic *A. oryzae* localized in the larval gut, decreased from the larval to the adult stage. However, they remained at relatively constant levels in the midgut of the adult mosquitoes for at least 10 days after emergence. Contrary to the view that gut sterilization occurs during metamorphosis from pupa to adult, some studies that mention trans-stadial transmission does occur from pupae to adults^48,58,70^. Our observations indicate that the microbiota clearance is not complete, with some of the larval gut microbes retained in the adult mosquito midgut^61,71,72^. Some bacteria such as *Pantoea stewartii, Stenotrophomonas maltophilia, Sphingobacterium multivorum* and *Chryseobacterium meningosepticum* remained in the midgut before and after metamorphosis and thus were not completely egested^17,73^. The 10 days persistence of *A. oryzae* –R in adult midguts in the current study contrasts with Rhiele et al.^74^ who found that *E. coli* was completely eliminated from the midgut of *An. stephensi* after just 96 h. *A. oryzae*-R and *E. coli* being^75^ very different organisms but perhaps other symbiont factors, including the different mosquito immune responses against bacteria and fungi^75,76^, could contribute to the differences between the two studies. In The bulk of midgut bacteria in the mosquito larval midgut is not transferred to the adult^47^, but are removed during metamorphosis, although there are some exceptions: *Serratia odorifera* is potentially maintained in larvae and trans-stadially transmitted to adult mosquitoes despite high environmental temperatures and strong proteolytic and hemolytic properties in the *Ae. aegypti* midgut^65^.

Thus, according to our results, *A. oryzae* may have some growth advantage in *Anopheles* larvae and adult midgut. This fungus has a wide pH tolerance range from pH 3 to pH 11 (data not shown). Likewise, larval gut pH ranges from 7.4 to 9 or higher^77,78^, and in the adult gut, pH is 7.4–7.8^77,79,80^. Since *A. oryzae*-R can colonize and survive in the larval midgut and are transferred through metamorphosis to the adult midgut, this pH tolerance of *A. oryzae* is a potential factor explaining its sustainability throughout the mosquito life cycle. Another possibility is that *A. oryzae* may be more resistant to any mosquito immune responses that may be triggered during both stages. Both these possibilities are interesting for further exploration of choice of organisms for refining paratransgenesis studies and applications.

*A. oryzae* were used to assess two potent anti-*Plasmodium* effector molecules, MP2 and EPIP. The expression and secretion of each fusion protein by the recombinant strains were verified by confocal microscopy and functional expression was shown by measuring the inhibition of oocyst formation in the mosquito midguts. Both molecules significantly inhibited malaria infections to *An. stephensi* at the oocyst stage. *A. oryzae* expressing MP2 was more effective than the strain expressing EPIP, reducing *P. berghei* oocyst intensity by 83% compared to 79.6%. Our initial experiments indicated that mixing the two strains expressing MP2 and EPIP reduced oocyst density to 11.5%, which was greater than the reduction achieved by MP2 or EPIP alone. In some experiments, mosquitoes fed with blood containing high *P. berghei* gametocyte numbers produced high oocyst intensities (mean > 200 oocysts), but even under these conditions, the inhibition of parasite development was highly significant and consistent.

In a previous study, anti-*Plasmodium* effector molecules secreted by recombinant *Pantoea agglomerans*, a bacterial symbiont of the mosquito, inhibited oocyst formation during malaria parasite development, in several species of *Anopheles*^30^. The transgenic fungi investigated here have a few advantages over these earlier studies. They are designated as safe in the food industry, therefore considered not harmful to humans or animals. *A. oryzae* is an effective laboratory model for paratransgenesis studies to evaluate various effector peptides/proteins as anti-parasite agents. Several aspects of our results support this claim; first, the pattern of localization suggests that *A. oryzae* localizes in the midgut, an important site the parasite development. Second, *A. oryzae* is transmitted from larvae to adult mosquitoes, while retaining their viability and capacity to secrete anti-parasite effector molecules^17,37^. According to our results, *A. oryzae* may be better adapted to the midgut environment than other agents such as mosquito bacteria. These results are consistent with proposals to select for paratransgenesis organisms that are compatible with the midgut environment^81^. Third, *A. oryzae* shows a stable association with larvae and adult mosquitoes as noted by its presence in all stages of *An. stephensi* through analysis using four different methods. Fourth, colonization experiments reveal that modified *A. oryzae*, acquired by larvae by consumption are capable of colonizing the gut of all inoculated larvae. Fifth, genetic modification of laboratory-reared fungi is easier than paratransgenic agents such as mosquito midgut bacteria and has already been used to control other vectors^13^.

Fungi are a viable and powerful model for evaluating and delivering proteins that block transmission and could be used to express multiple transgenes with different mechanisms of action to reduce the likelihood of developing resistance. Accordingly, numerous effector peptides or proteins can be engineered into *A. oryzae,* and the collection of effector genes can be modified to maximize efficacy in combating malaria or other vector-borne diseases. We conclude that trans-stadial transmission of transgenic *A. oryzae* occurs from the larval to the adult stage of *An. stephensi*. Therefore, in a paratransgenic approach, it may be possible to spread the transgenic fungi in breeding sites where mosquito larvae can be exposed to the fungal spores. Recombinant *A. oryzae*, modified with two different peptides (MP2 and EPIP) inhibits oocyst formation of *P. berghei* in the midgut of adult *An. stephensi* and could be used as carriers of additional effector molecules that can function as anti-parasite agents. Importantly, paratransgenesis is compatible with current mosquito control tools and even with genetically modified mosquitoes.

*A. oryzae* naturally occurring in the soil and environment could be used in integrated vector control approaches, and this method is proving to be an environmentally friendly approach to controlling larvae in the natural habitats themselves. Fungal biopesticides are already approved for agricultural use in a number of African countries along with chemical insecticides^82^. The narrow host range of the transgenic fungi limits the potential hazards to non-target insects. For *Anopheles* larvae in an aquatic habitat, the impact on beneficial insects such as bees would be unlikely. There are limited data concerning the effects of biopesticides on non-target species in mosquito breeding sites. Extracts of *Aspergillus terreus* affected swimming behavior of *Artemia*, but these extracts were neurotoxic to mosquitoes and *Artemia* is not sympatric with mosquito larvae^83^. Similarly, extracts of *Asperillus flavus* affected the predatory mosquito, *Toxorhynchites splendens* but at higher concentrations than affecting the target, *Ae. aegypti*^84^. Studies on effects of live entomopathogenic fungi in aquatic conditions limited, representing a major gap in this research field, but combining fungi and predatory mosquitoes may give a more optimum control outcome^85^. Given the diversity and ephemeral nature of *Anopheles* breeding sites, characterizing the majority of breeding sites would be unnecessary and also impractical in a control program^86^, so any biopesticide approaches will need to consider effectiveness in different environmental conditions. While the results of a single case study should be treated with appropriate caution to avoid over-interpretation, we believe we have demonstrated that experimental studies of the role of fungi in natural insect communities are feasible and have the potential to yield promising insights.

## Supplementary Information

Supplementary Information 1.

Supplementary Information 2.

## Data Availability

All data generated or analyzed during this study are included in this published article and its supplementary information files.
