# Supplementary material for: A recombinant Aspergillus oryzae fungus transmitted from larvae to adults of Anopheles stephensi mosquitoes inhibits malaria parasite oocyst development
Source: Sci Rep. 2023 Jul 27;13:12177. doi: 10.1038/s41598-023-38654-0 (PMC10374630; doi:10.1038/s41598-023-38654-0)
Supplement: Supplementary file 2 — Supplementary Information 2. [file 41598_2023_38654_MOESM2_ESM.pdf]

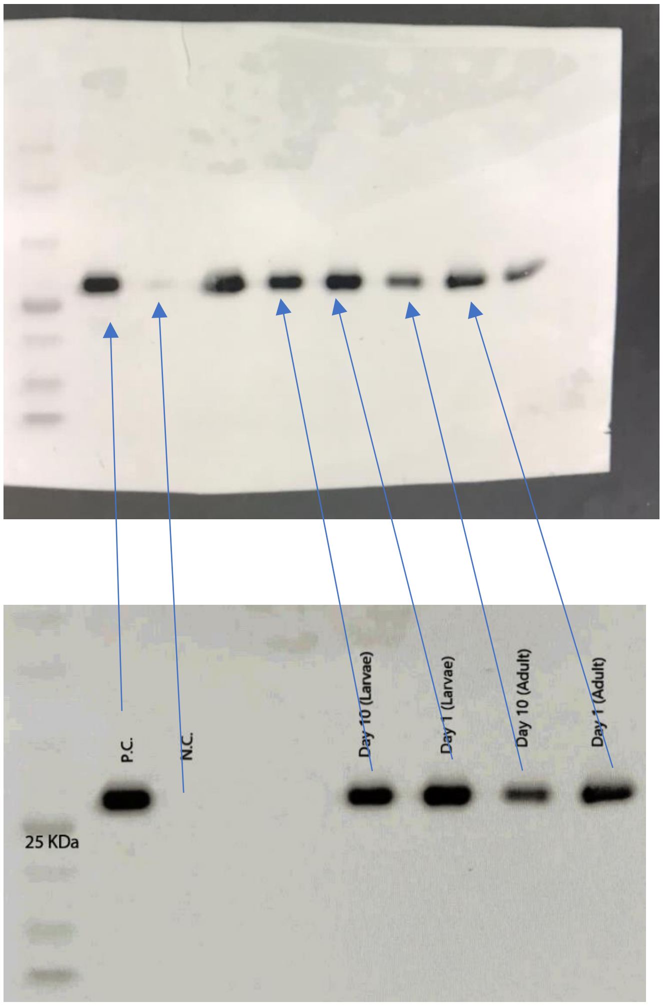

Figure S1. Western blot analysis of protein secretion. Aliquots of equal protein concentration were analyzed by Western blotting using the GFP monoclonal antibody. *A. oryzae*-R was inoculated into *An. stephensi* L_1 larvae via water for 24 hours, except for the negative control, which was inoculated with wild-type *A. oryzae* (N.C.). The concentrated supernatant of *A. oryzae*-R served as the positive control (P.C.), on the first and tenth day at the larval stage and on day one and day ten at the adult stage. Shown are the bands for the cleaved forms of GFP (28 kDa). Top = original western blot, bottom = cropped western blot (also shown as Figure 5 in main body of the paper).

(i (ii

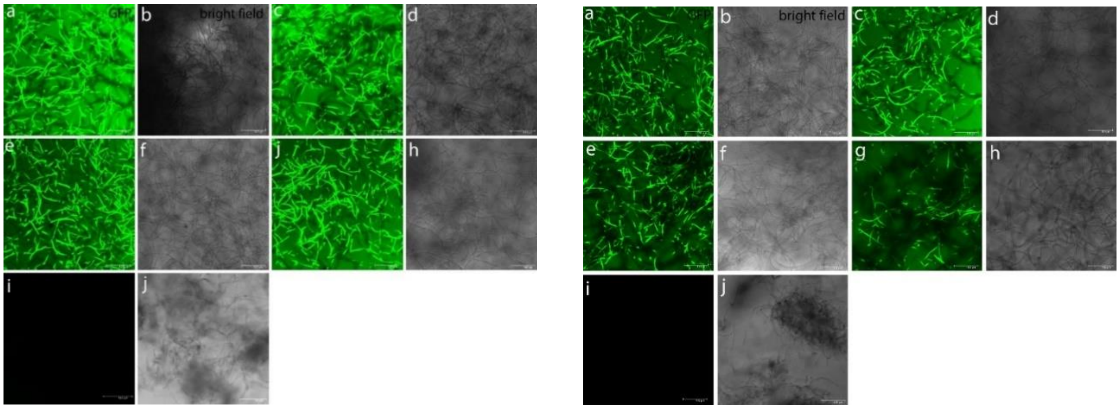

Figure S2. *A. oryzae*- R isolated from the larvae (**i**) and the adult mosquito midgut (**ii**). The isolates with GFP expression were visualized by confocal microscopy, (i,j) The control group that was not inoculated with any fungus, (a,b) day first, (c,d) fourth (e,f) seventh, and (g,h) tenth. Scale bar, 100 µm.

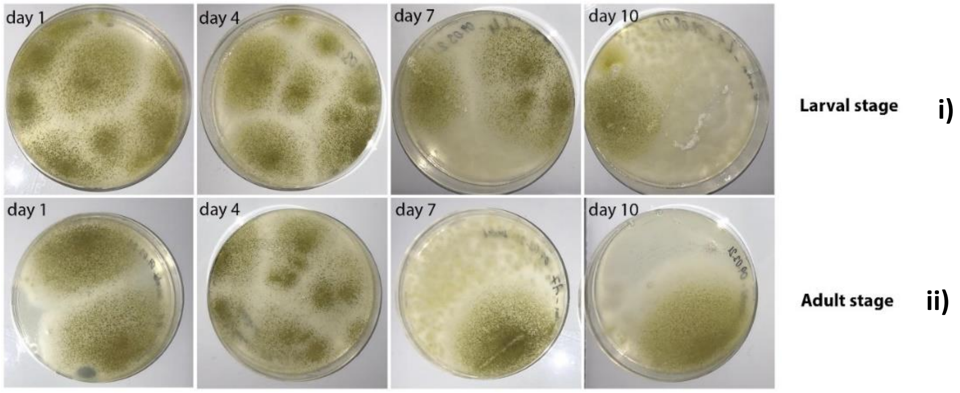

Figure S3. *A. oryzae*-R was introduced into *A. stephensi* first instar. The larva and the adult mosquito's midguts were inoculated at four time points (days 1, 4, 7, and 10) post inoculation in the larval stage and at four time points (days 1, 4, 7, and 10) post emergence in the adult stage, on CD agar plates containing ampicillin and kanamycin separately. (i) Larval stage, (ii) Adult stage (The experiments were repeated three times with similar results).
